# E2F1-Induced lncRNA BAIAP2-AS1 Overexpression Contributes to the Malignant Progression of Hepatocellular Carcinoma via miR-361-3p/SOX4 Axis

**DOI:** 10.1155/2021/6256369

**Published:** 2021-09-25

**Authors:** Yan Yang, Hong Ge, De-qing Li, Ai-xia Xu

**Affiliations:** ^1^Department of Clinical Laboratory, The Affiliated Lianyungang Hospital of Xuzhou Medical University, The First People's Hospital of Lianyungang, Haizhou District, Lianyungang, China; ^2^Department of Surgery, The Qingdao Eighth People's Hospital, Qingdao, Shandong, China; ^3^Department of Radiology, Weifang Municipal Hospital, Weifang, Shandong, China; ^4^Department of Pharmacy Intravenous Admixture, Weifang People's Hospital, Weifang, Shandong, China

## Abstract

Currently, plenty of researches have revealed that long noncoding RNAs (lncRNAs) can act as crucial roles during the progression of various tumors, including hepatocellular carcinoma (HCC). Here, we measured the expression of lncRNA BAIAP2 antisense RNA 1(BAIAP2-AS1) as well as its contribution to the developments of HCC. In this study, the expressions of BAIAP2-AS1 and SOX4 were distinctly upregulated in HCC cells and tissues, and high BAIAP2-AS1 may be a novel biomarker for HCC. E2F1 activated BAIAP2-AS1 expression. The silence of BAIAP2-AS1 inhibited the proliferation and metastasis of HepG2 and PLC5 cells. Assays for relationship verification showed that BAIAP2-AS1 regulated the expression of SOX4 and miR-361-3p. Rescue experiments further confirmed the positive interaction between miR-361-3p and BAIAP2-AS1 as well as between miR-361-3p and SOX4. Overall, BAIAP2-AS1 modulated the miR-361-3p/SOX4 axis to promote the development of HCC. Thus, our study offers a potential therapeutic target for treating HCC.

## 1. Introduction

Hepatocellular carcinoma (HCC) is a primary malignancy of the liver, now ranking the sixth most common malignant tumor and causing an estimated over half a million deaths annually [1, 2]. It is more predominant in parts of Asia and Central Africa, and approximately 50 percent of all new cases are diagnosed in China [3]. Despite several advances in diagnostic strategies, chemotherapy, radiotherapy, and surgery, the survival time of many HCC patients remains poor due to drug resistance, tumor relapse, and late diagnosis [4, 5]. Thus, the molecular mechanisms regarding HCC initiations and developments deserve further exploration.

The vast majority of human transcriptome could be classified as noncoding RNAs based on the advancements of high-throughput RNA sequencing technology [6]. As a class of noncoding RNAs, long noncoding RNAs (lncRNAs) have a length of >200 nucleotides (nt) [7]. Multiple model organisms have been used for the annotation of lncRNAs, and the results suggest that they are often expressed in a spatial- and temporal-specific pattern [8, 9]. In recent years, emerging studies have suggested lncRNA involvements in diverse physiological and pathological processes, such as programmed cell death, cellular growth, transport through nuclear pore, stem cell pluripotency, and developments, via serving as epigenetic regulators at transcriptional, posttranscriptional, or epigenetic levels [10, 11]. Growing evidence also confirms that lncRNAs exhibit important regulatory effects in the progression of various tumors via a series of complex mechanisms [12, 13]. However, the potential functions of many lncRNAs in HCC development and metastasis have not been elucidated.

In this research, lncRNA BAIAP2 antisense RNA 1 (BAIAP2-AS1) was identified as a novel HCC-related lncRNA. It was observed to be highly expressed in HCC. Then, for the first time, the clinical significance and tumor-related functions of BAIAP2-AS1 in HCC were investigated. This research gained new insights into the metastatic mechanism of HCC and provided HCC treatment with a promising therapeutic target.

## 2. Materials and Methods

### 2.1. Clinic Samples

Totally, 10 patients receiving surgery in the Weifang People's Hospital between July 2019 and August 2020 were adopted to obtain 10 paired HCC and corresponding adjacent normal specimens. Before sample collection, local or systemic treatments were not applied to all patients. All specimens were snap-frozen and stored at -80 for the extraction of total RNAs. Two experienced pathologists identify the specimen types. Inform consent of all patients was obtained before the surgery, together with the approval of the Ethics Committee of the Weifang People's Hospital for the study.

### 2.2. Cell Lines and Cell Transfection

The HCC cell lines HCCLM3, Huh7, HepG2, and PLC5 and the normal liver cell line LO2 were purchased from Shanghai Cell Bank (Shanghai, China). All cells were grown in DMEM (Procell, Wuhan, Hubei, China) containing 10% FBS (Cyagen Technology, Taicang, Jiangsu, China) and 100 *μ*g/ml streptomycin and penicillin.

Short hairpin RNAs (shRNAs) against BAIAP2-AS1 were ligated into pGPU6/GFP/Neo1 vectors (sh-BAIAP2-AS1-1: CCGGCAGTAACCAGAAAGTTCCAGACTCGAGTCTGGAACTTTCTGGTTACTGTTTTTTG; sh-BAIAP2-AS1-2: CCGGCACTTGTAATCAGTAACCAGACTCGAGTCTGGTTACTGATTACAAGTGTTTTTTG) (Yunzhou Technology, Guangzhou, Guangdong, China), and plasmid with non-targeting sequences was applied as a negative control (sh-NC). E2F1 small interfering RNAs (si-E2F1-1: 5′-CCACTCCACCTAACCATAGTCCACT-3′; si-E2F1-2: ACCCTATTCATCACGTCCCACCACT) and negative control (NC) siRNA were purchased from Origene (Haidian, Beijing, China). miRNA-361-3p mimics (5′-TCCCCCAGGTGTGATTCTGATTT-3′), miRNA-361-3p inhibitors (5′-ACTTCGTCTCATGCACTATTTACA-3′), NC mimics (5′-TAACACGCATTATTCACCAGGCACA-3′), and NC inhibitors (5′-TCAACAACTGTGTCTCACCTGTCA-3′) were obtained from Weizhen Biology (Jinan, Shandong, China). Lipofectamine 2000 reagent (Invitrogen) was used to conduct cell transfection based on instructions of the manufacturer.

### 2.3. Bioinformatics Analysis

The binding sites among BAIAP2-AS1, miR-361-3p, and SOX4 were predicted on StarBase (http://starbase.sysu.edu.cn/index.php). JASPAR was applied for the prediction of the binding site of E2F1 in the BAIAP2-AS1 promoter region (http://jaspar.genereg.net/). The number of promoters was predicted by software including Promoter 2.0 [14], FPROM [15], and NNPP [16]. The relative profile score threshold was 80%. “GEPIA” analyzed the expression of BAIAP2-AS1 and SOX4 and their clinical significance (http://gepia.cancer-pku.cn/).

### 2.4. RNA Extraction and Quantitative Real-Time PCR (RT-qPCR)

For the collection of total RNA from specimens and cells, TRIzol reagent which was purchased from Life Technologies (Haidian, Beijing, China) was used. Reverse EasyScript One Step gDNA Removal and cDNA Synthesis SuperMix (TAKALA, Hangzhou, Zhejiang, China) were applied to synthesize first-strand cDNA. ABI 7300 Sequence Detection System (Nanjing, Jiangsu, China) was applied for the performance of RT-qPCR. GAPDH or U6 snRNA was used for the endogenous control. Moreover, 2^−*ΔΔ*Ct^ methods were used to calculate the fold change in gene expressions. Primers were designed by Primer Express 3.0.  Table 1 lists the sequences.

### 2.5. Cell Counting Kit-8 (CCK-8)

The CCK-8 solution (Dojindo, Gaithersburg, MD) was used for the cellular proliferation assays. First, cells were seeded in triplicate into 96-well plates at a concentration of 3 × 10^3^ cells/well. Then, 15 *μ*l/well of CCK-8 solution was applied for the treatments of the collected cells during the ultimate 4 h of culture. A microplate reader was applied to measure optical density.

### 2.6. Colony Formation Assays

Cells were plated in six-well plates at a density of 600 cells per well and were cultured with RPMI 1640 (Sigma-Aldrich, Shenzhen, Guandong, China) supplemented with ten percent FBS (Cyagen Technology, Taicang, Jiangsu, China) for ten days. When the incubation period finished, PBS was used to wash the cells, which then were fixed in methanol and dyed by crystal violet (Tsbiochem, Jingan, Shanghai, China). Three independent experiments were performed.

### 2.7. EdU Staining

The cell culture medium was added by EdU assay kit for three hours. Then, a Cell-Light™ EdU Apollo®^489^ in vitro Imaging Kit was applied for the stain of the cells. DAPI staining was conducted on the cell nucleus without light. An inverted fluorescence microscope was used for the observation.

### 2.8. Invasion Assays

The invasive potentials of cells were measured by the use of transwell assays. The procedures were performed as described previously [17].

### 2.9. Subcellular Fractionation

A PARIS kit (Life Technologies, Shenzhen, Guangdong, China) was used for nuclear and cytosolic fraction separation under the manufacturer's instructions [18].

### 2.10. Biotin RNA Pull-Down Assays

Biotin-labeled sense or antisense oligos of BAIAP2-AS1 were incubated with HepG2 and PLC5 cell lysate for one hour. Streptavidin-coupled agarose beads (Invitrogen, Carlsbad, USA) were applied for the pull-down of the complex. Sense probes included 5′-(biotin-) ACTTGCATGGCTGCACGCATCCTCATAAGACG-3′, 5′-(biotin-) CATCCAACCTCCAGAGACACCTGCGCCACA-3′, and 5′-(biotin-) AACCTCACGCACGTCGATCGTCACCACC-3′. Antisense probes comprised 5′-(biotin-) CCACTACTGACCTACGTATCCTTCAGCCACCC-3′, 5′-(biotin-) ACCACTCCCTCAGTCAGTCCACGTCACAG-3′, and 5′-(biotin-) ACTCGTCCACTGGTCACACCACTGCAT3′. The isolated RNAs were transcribed into cDNA, and RT-qPCR measured the levels of BAIAP2-AS1 and miR-361-3p.

### 2.11. Chromatin Immunoprecipitation (ChIP) Assays

ChIP assays were performed with a Magna ChIP Kit (Millipore, Hangzhou, Zhejiang, China). To generate DNA-protein cross-links, formaldehyde was applied to HepG2 and PLC5 cells. Chromatin fragments of 200-300 bp were generated by sonication of cell lysates. It was followed by immunoprecipitation of lysates with specific antibodies. In addition, IgG was used as the control. For the examination of the precipitated chromatin DNA, RT-qPCR was carried out.

### 2.12. Luciferase Reporter Assays

To predict the E2F1-binding site in the promoter of BAIAP2-AS1, the JASPAR database was searched [19]. Then, we synthesized the diverse fragment sequences of the promoter of BAIAP2-AS1. The vectors generated by inserting the above sequences into the pGL3-basic vector were cotransfected with E2F1 expression plasmid into HepG2 and PLC5 cells.

We cotransfected HepG2 and PLC5 cells with pmir-GLO-BAIAP2-AS1-WT, pmirGLO-BAIAP2-AS1-Mut, pmirGLO-SOX4-3′UTR-WT, pmirGLO-SOX4-3′UTR-Mut reporter plasmids, NC mimics, and miR-361-3p mimics. Under the application of a dual-luciferase reporter assay system (Promega, Hangzhou, Zhejiang, China), the relative luciferase activity was measured. Then, we normalized the collected data to Renilla luciferase activity.

### 2.13. Western Blot Assays

A 1× sodium dodecyl sulfate buffer was used to prepare total cell lysates. After being separated by SDS-PAGE, identical quantities of proteins were placed onto PVDF membranes. The incubation of blots was conducted first with antibodies specific for human GAPDH (ab8245), E2F1 (ab4070), SOX4 (ab243739), E-cadherin (ab233611), vimentin (ab92547), or N-cadherin (ab76011) and then with HRP-conjugated second antibody. TTBS was used to wash the blots for six times. Then, the Beyo ECL Plus reagent was applied to develop the blots, and the Gel Imaging System was used to record the images. Abcam (Pudong, Shanghai, China) provided all antibodies. Normalization of data was conducted for expressions of GAPDH (Rabbit anti-GAPDH).

### 2.14. Statistical Analysis

The SPSS statistical software package (standard version 18.0, SPSS Inc., Chicago, IL, USA) was applied to statistical analysis, and mean ± SD was used as a form of values. Unless otherwise stated, the statistical significance for comparisons of 2 or more groups was identified by one-way ANOVA and Student's *t*-test. *P* < 0.05 was considered statistically significant.

## 3. Results

### 3.1. BAIAP2-AS1 Was Upregulated in HCC

**Figure 1:**
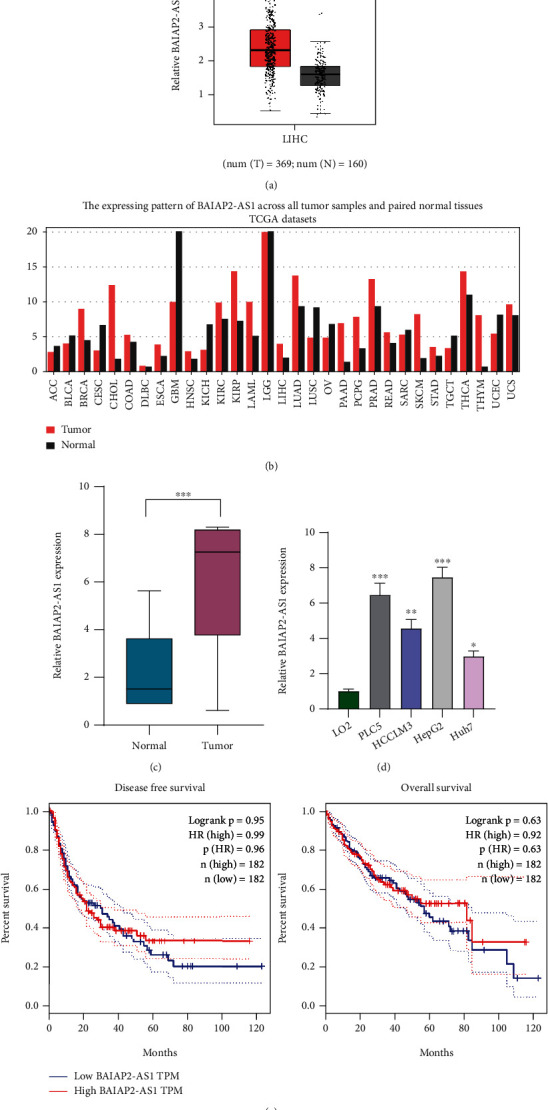
The levels of BAIAP2-AS1 in HCC patients and its clinical significance. (a) The levels of BAIAP2-AS1 in HCC and normal liver specimens based on TCGA datasets (tumor: *n* = 369, normal: *n* = 160). (b) Pan-cancer expression of BAIAP2-AS1 using TCGA datasets. (c) RT-qPCR assay was applied to analyze BAIAP2-AS1 expression in fresh HCC specimens and matched control tissues. (d) The expression of BAIAP2-AS1 in LO2 cells and HCC cell lines Huh7, HCCLM3, HepG2, and PLC5. (e) The association between the dysregulated BAIAP2-AS1 and clinical survivals by analyzing TCGA datasets (*n* = 364). ^∗^*p* < 0.05,  ^∗∗^*p* < 0.01, and^∗∗∗^*p* < 0.001.

We searched GEPIA which further showed BAIAP2-AS1 levels displayed an increasing trend in HCC specimens (*n* = 369) compared with normal liver specimens (*n* = 160) (Figure 1(a)). Moreover, Pan-cancer assays revealed that overexpression of BAIAP2-AS1 was a frequent event in many types of tumors (Figure 1(b)). To confirm the above results, we performed RT-qPCR in our cohort, finding that compared to matched nontumor specimens, the expressions of BAIAP2-AS1 were obviously decreased in HCC specimens (Figure 1(c)). We also observed high expression of BAIAP2-AS1 in four HCC cell lines compared to LO2 cells (Figure 1(d)). Besides, we observed that BAIAP2-AS1 exhibited a higher expression in HepG2 and PLC5 cells than in the other two cells. Thus, we chose HepG2 and PLC5 cells for further functional experiments. Then, we used GEPIA to analyze the influence of BAIAP2-AS1 expression on survival time. As shown in Figure 1(e), we did not observe the association between BAIAP2-AS1 expression and OS and DFS (*P* > 0.05).

### 3.2. E2F1 Activates BAIAP2-AS1 Transcription in HCC Cells

**Figure 2:**
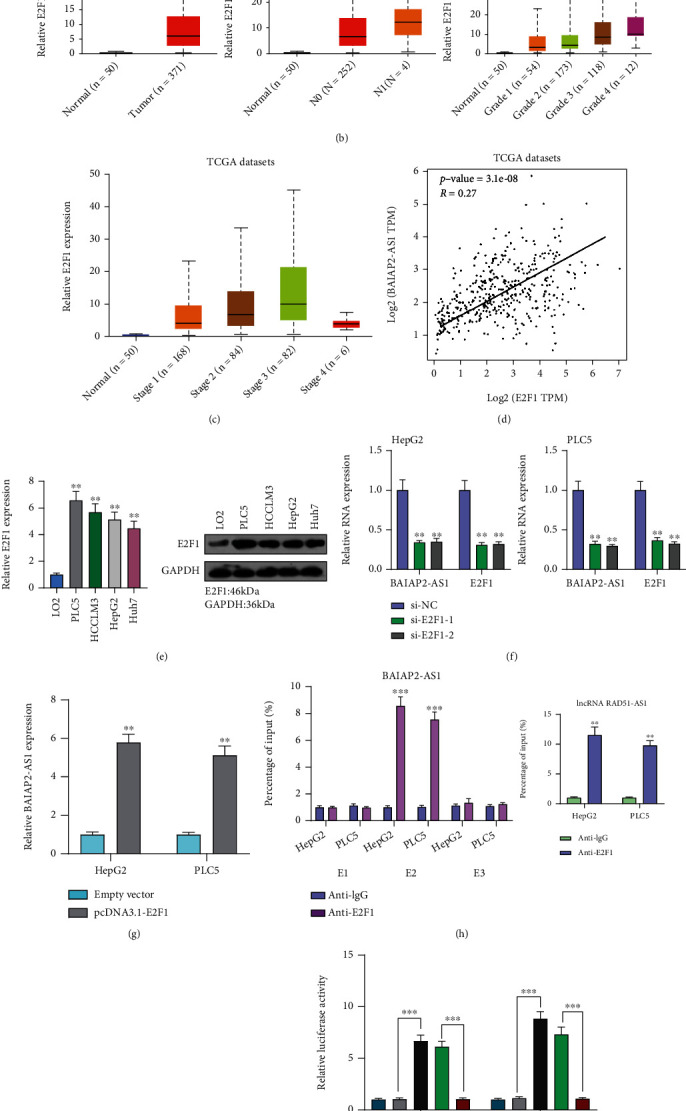
E2F1 activates BAIAP2-AS1 expression in HCC cells. (a) E2F1 binding site prediction in the BAIAP2-AS1 promoter region by the use of JASPAR. (b, c) The expression pattern of BAIAP2-AS1 in HCC by analyzing TCGA datasets. (d) The correlation between E2F1 and BAIAP2-AS1 expressions in HCC samples using TCGA datasets. (e) Expression level of BAIAP2-AS1 was detected in four HCC cells using RT-qPCR and Western blot. (f) The expression of BAIAP2-AS1 and E2F1 in HepG2 and PLC5 cells transfected with si-E2F1-1 or si-E2F1-2. (g) Overexpression of E2F1 resulted in the upregulation of BAIAP2-AS1. (h) ChIP-qPCR analysis of E2F1 occupancy in the BAIAP2-AS1 promoter in HepG2 and PLC5 cells. (i) Construction of the luciferase reporter vector. (j) Dual-luciferase reporter assays were applied to examine the combination between BAIAP2-AS1 and E2F1. ^∗∗^*p* < 0.01 and ^∗∗∗^*p* < 0.001.

Substantial researches have reported lncRNA dysregulation in cancers; nonetheless, the factors regulating these molecules' misregulation remain unclear [20, 21]. More and more researchers found proof for the contribution of some crucial transcription factors to lncRNA dysregulation in human cancer cells [22, 23]. To examine the mechanism of BAIAP2-AS1 upregulation in HCC, this research explored the promoter region of BAIAP2-AS1 by online bioinformatics software programs JASPAR, revealing six potential sites of E2F1 binding (Figure 2(a)). Then, the expressing pattern of E2F1 in HCC was determined by UALCAN, finding increased E2F1 expression in HCC specimens, especially in those with advanced stages (Figures 2(b) and 2(c)). Based on TCGA datasets, we also observed that E2F1 expression is positively associated with E2F1 in HCC specimens (Figure 2(d)). We also showed the increased levels of E2F1 in five HCC cell lines compared with LO2 cells (Figure 2(e)). To study whether the dysregulation of E2F1 may influence the expression of BAIAP2-AS1, we overexpressed or downregulated the expression of E2F1 and performed RT-qPCR which showed that BAIAP2-AS1 levels were distinctly decreased after the knockdown of E2F1, while E2F1 overexpression displayed an opposite result (Figures 2(f) and 2(g)). Clear E2F1-binding activity was implied by subsequent ChIP assays on the endogenous BAIAP2-AS1 promoter region around E2 (Figure 2(h)). Moreover, E2F1 did not bind to the sites except for E2 (-1424 bp), as revealed by luciferase reporter assays (Figures 2(i) and 2(j)).

### 3.3. BAIAP2-AS1 Knockdown Suppressed the Proliferation and Metastasis of HCC Cells

**Figure 3:**
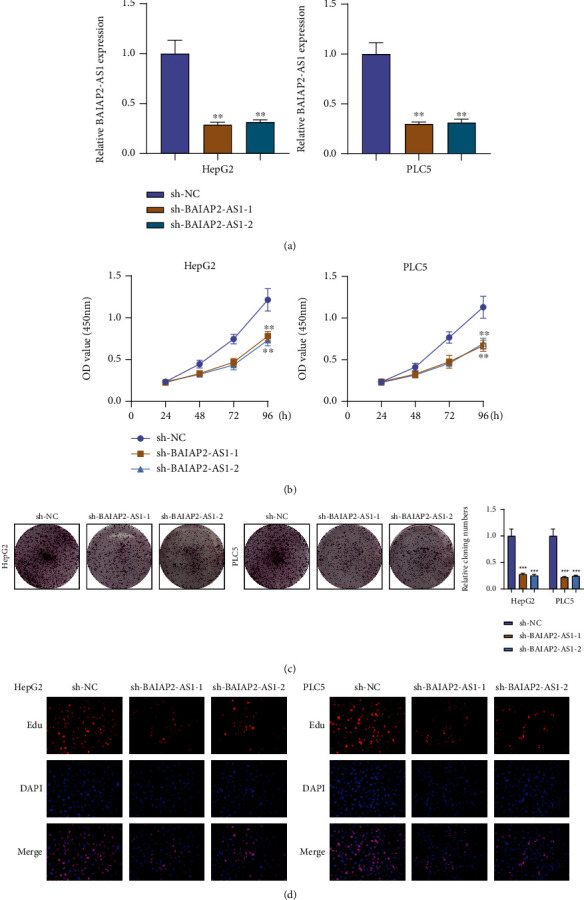
Effect of BAIAP2-AS1 knockdown on cell proliferation. (a) BAIAP2-AS1 levels were decreased in HepG2 and PLC5 cells transfected with sh-BAIAP2-AS1-1 or sh-BAIAP2-AS1. (b) CCK-8 test of cell proliferation in HCC cells. (c) Clone formation assays were performed to examine cell vitality after transfection. (d) Edu proliferation assays determined cell proliferation ability of HepG2 and PLC5 cells after transfection. ^∗∗^*p* < 0.01 and ^∗∗∗^*p* < 0.001.

**Figure 4:**
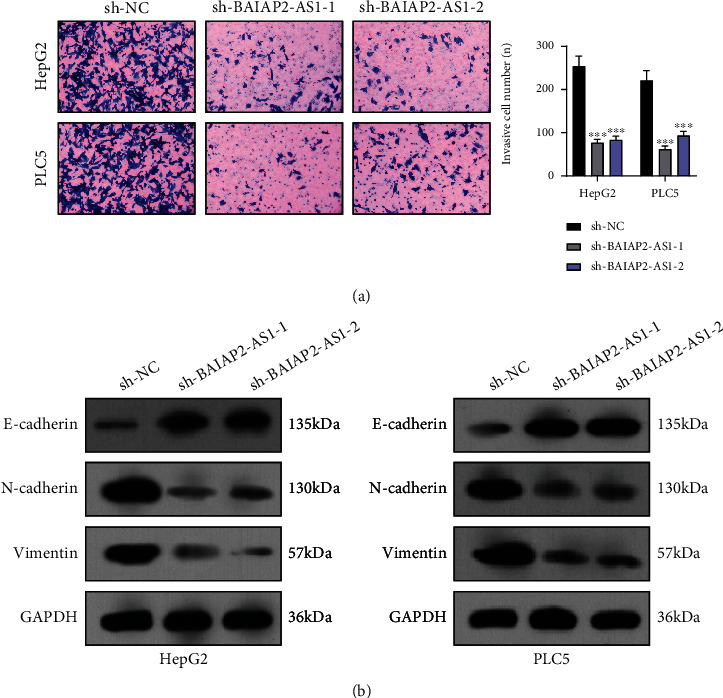
BAIAP2-AS1 knockdown inhibited tumor metastasis. (a) Detecting cell invasion by transwell invasion assays. (b) The protein levels of EMT markers (N-cadherin, E-cadherin, and vimentin) were assessed using Western blotting. ^∗∗^*p* < 0.01 and ^∗∗∗^*p* < 0.001.

For examining the biological influence of BAIAP2-AS1 on HCC growth, we transfected HepG2 and PLC5 cells with sh-BAIAP2-AS1-1, BAIAP2-AS1-2 or sh-NC. RT-qPCR confirmed a distinct reduction in the expression level of BAIAP2-AS1 in HepG2 and PLC5 cells treated with sh-BAIAP2-AS1-1 and BAIAP2-AS1-2 (Figure 3(a)). Consequently, the results of CCK-8 assays revealed that cell viability was significantly decreased in sh-BAIAP2-AS1-1 and BAIAP2-AS1-2 transfected cells than in sh-NC transfected cells (Figure 3(b)). Also, the clonogenic survival of HepG2 and PLC5 cells was shortened by BAIAP2-AS1 knockdown as found from colony formation assays (Figure 3(c)). Edu assays also confirmed the BAIAP2-AS1 knockdown suppressed HCC cells' proliferation (Figure 3(d)). Furthermore, cell invasion abilities were investigated by transwell invasion. Compared to the sh-NC group, the invasion capacities of HepG2 and PLC5 cells transfected with sh-BAIAP2-AS1-1 or sh-BAIAP2-AS1-2 were significantly decreased (Figure 4(a)). To explore the mechanisms involved in the effects of BAIAP2-AS1 on metastasis capacities of HCC cells, we observed that silence of BAIAP2-AS1 promoted the expression of E-cadherin while suppressing the expression of N-cadherin and vimentin in HepG2 and PLC5 cells (Figure 4(b)). Based on the above results, BAIAP2-AS1 was concluded as a tumor promoter in HCC.

### 3.4. BAIAP2-AS1 Directly Interacts with miR-361-3p and Represses Its Expression

**Figure 5:**
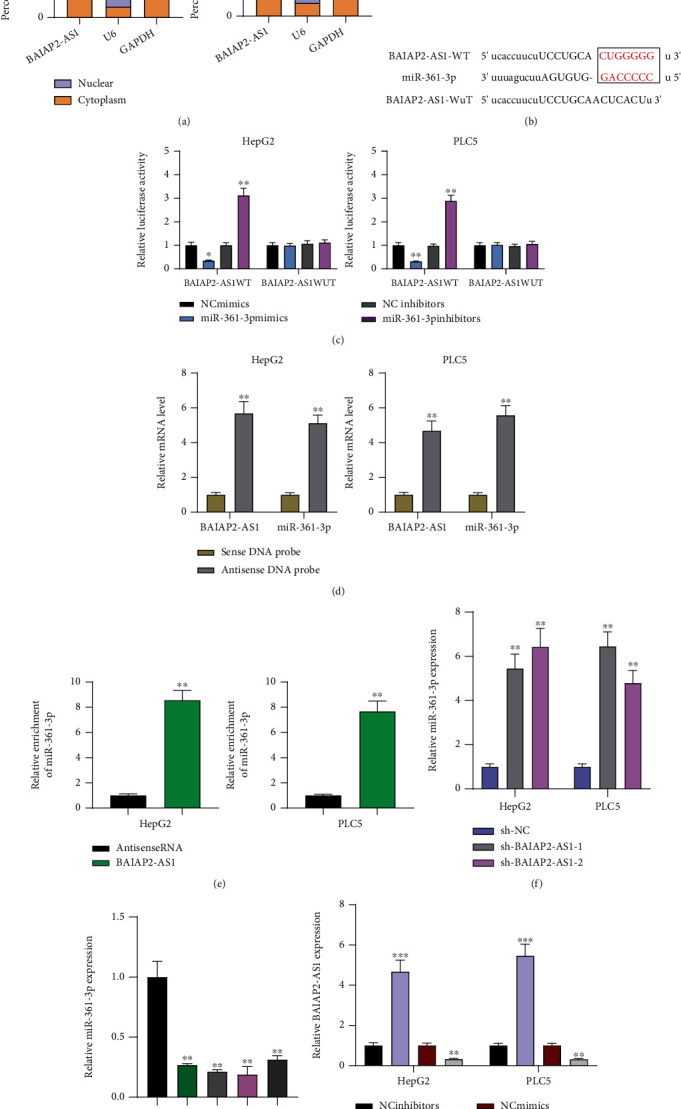
Regulation relationship between BAIAP2-AS1 and miR-361-3p. (a) Relative BAIAP2-AS1 expressions in cytosolic and nuclear fractions of HepG2 and PLC5 cells. (b) Diagram of the BAIAP2-AS1 putative binding sites and corresponding mutant sites in BAIAP2-AS1 mRNA sequences. (c) Luciferase activity in HepG2 and PLC5 cells cotransfected with miR-361-3p mimics or miR-361-3p inhibitors and luciferase reporters containing BAIAP2-AS1-WT or BAIAP2-AS1-MT. (d) Biotin-coupled sense or antisense DNA probes targeting BAIAP2-AS1 were incubated with HepG2 and PLC5 cells lysate to pull down RNAs which were further analyzed using RT-qPCR for the amounts of BAIAP2-AS1 and miR-361-3p. (e) Biotin-labeled BAIAP2-AS1 RNA and antisense RNA were incubated with HepG2 and PLC5 cell lysate to pull down RNAs. (f) Dysregulation of miR-361-3p expression in BAIAP2-AS1 knockdown HepG2 and PLC5 cells. (g) RT-qPCR analysis of miR-361-3p expression in four HCC cells and LO2 cells. (h) RT-qPCR assays of the levels of BAIAP2-AS1 in HepG2 and PLC5 cells after overexpression or knockdown of miR-361-3p. ^∗∗^*p* < 0.01 and ^∗∗∗^*p* < 0.001.

For exploring how BAIAP2-AS1 promoted the malignant phenotypes of HCC cells, its subcellular localization was examined under the assumption of the dependence of one lncRNA's function on its subcellular distribution [24]. As shown in Figure 5(a), subcellular fractionation suggested that BAIAP2-AS1 was mainly expressed in the cytoplasm. As found by StarBase v2.0 software, BAIAP2-AS1 may be a possible target of BAIAP2-AS1 (Figure 5(b)). Luciferase reporter assay demonstrated that BAIAP2-AS1-WT and miR-361-3p mimic cotransfection memorably depressed luciferase activity (Figure 5(c)). The similar findings were also observed when BAIAP2-AS1-MUT and miR-361-3p inhibitor cotransfection was performed. Furthermore, BAIAP2-AS1 antisense probe pulled down both miR-361-3p and BAIAP2-AS1 RNA (Figure 5(d)). In addition, miR-361-3p from HepG2 and PLC5 cells lysate was also enriched by full-length BAIAP2-AS1 RNA (Figure 5(e)). Knockdown of BAIAP2-AS1 was observed to promote miR-361-3p's expression (Figure 5(f)). Then, its levels in HCC cells and specimens were explored, finding obviously higher BAIAP2-AS1 expression levels in HCC cells (Figure 5(g)). Finally, the downregulation of miR-361-3p was found to cause a higher expression level of BAIAP2-AS1, while its overexpression had an opposite result (Figure 5(h)).

### 3.5. BAIAP2-AS1 Regulates miR-361-3p to Modulate SOX4 in HCC Cells

**Figure 6:**
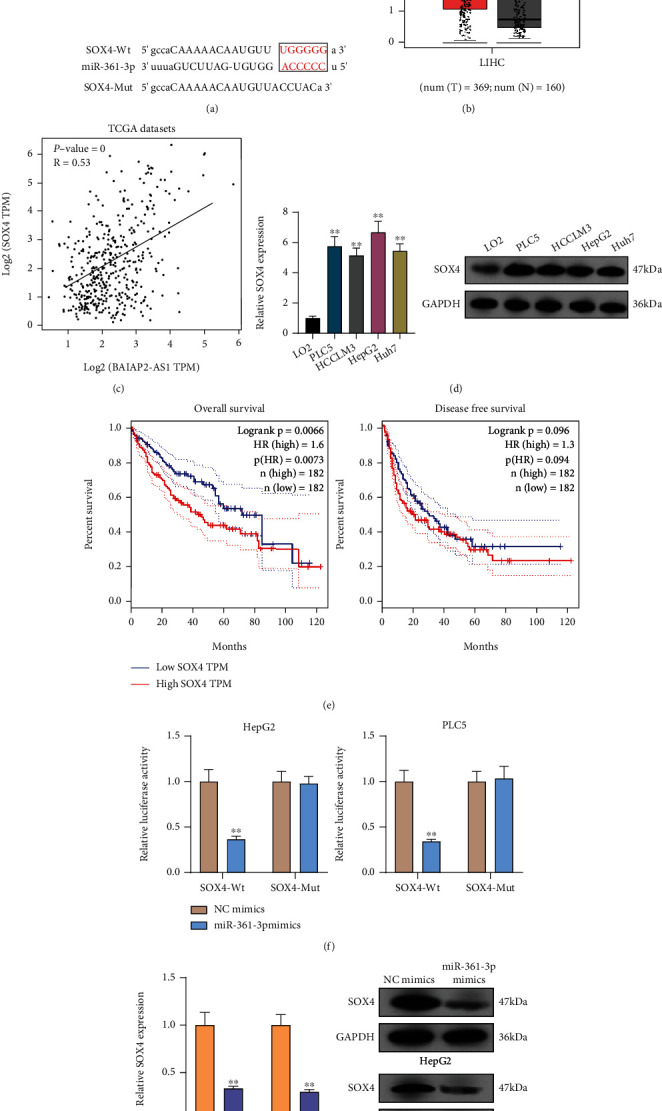
SOX4 is directly targeted by miR-361-3p. (a) Sequence alignment of miR-361-3p and 3′UTR of SOX4 using StarBase 2.0. (b) The overexpression of SOX4 in HCC specimens compared to normal tissues by analyzing TCGA datasets. (c) The expressions of SOX4 were positively correlated with BAIAP2-AS based on TCGA datasets. (d) The expression of miR-361-3p was measured in five HCC cell lines and LO2 cells by RT-qPCR. (e) Survival assays of 362 HCC patients according to SOX4 expression based on TCGA datasets. (f) Luciferase reporter assays were performed to verify the binding of miR-361-3p in 3′UTR of SOX4. (g) The HepG2 and PLC5 cells transfected with miR-361-3p mimics exhibited an increased expression of SOX4. ^∗∗^*p* < 0.01.

**Figure 7:**
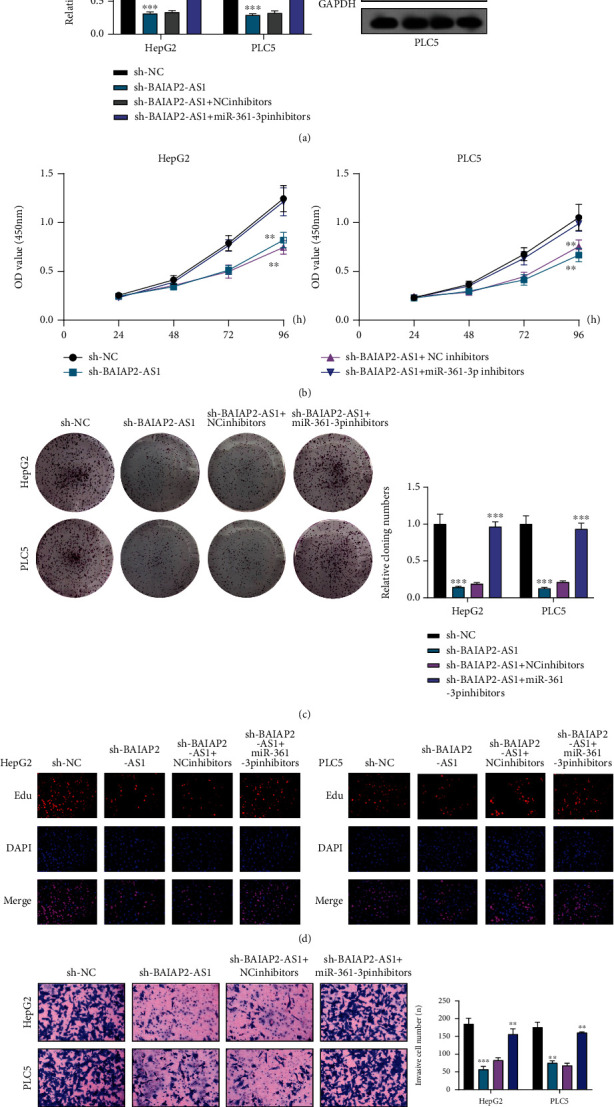
The regulation of BAIAP2-AS1 on miR-361-3p/SOX4 was studied. (a) RT-qPCR and Western blot determined the expression of SOX4 in HepG2 and PLC5 cells transfected with sh-NC, sh-BAIAP2-AS1, sh-BAIAP2-AS1/NC inhibitors, or sh-BAIAP2-AS1/miR-361-3p inhibitors. (b) CCK-8 assays. (c) Colony formation assay. (d) Edu assays. (e) Transwell assay in HepG2 and PLC5 cells transfected with sh-NC, sh-BAIAP2-AS1, sh-BAIAP2-AS1/NC inhibitors, or sh-BAIAP2-AS1/miR-361-3p inhibitors. ^∗∗^*p* < 0.01 and ^∗∗∗^*p* < 0.001.

**Figure 8:**
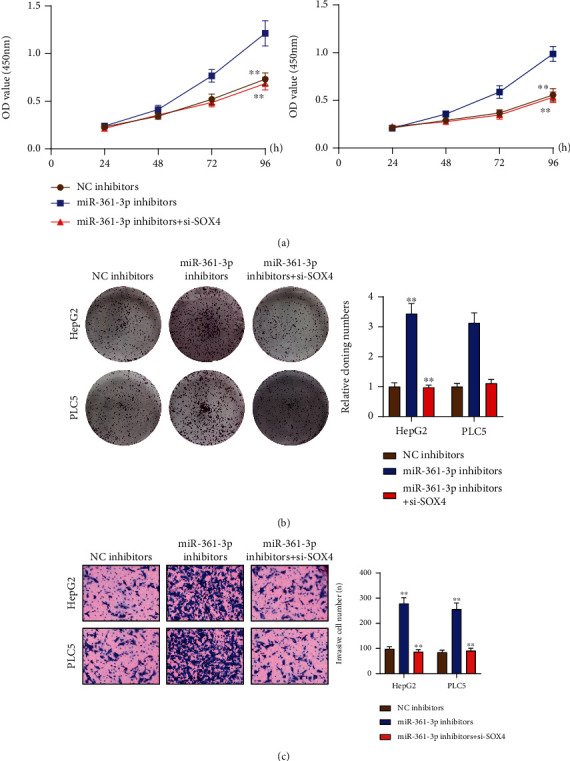
SOX4 knockdown could reverse the enhanced proliferation and invasion ability of HepG2 and PLC5 cells caused by miR-361-3p inhibition. (a) CCK-8 assays, (b) colony formation assays, and (c) transwell assays in HepG2 and PLC5 cells transfected with NC inhibitors, miR-361-3p inhibitors, or miR-361-3p inhibitors+si-SOX4. ^∗∗^*p* < 0.01.

The influence of BAIAP2-AS1 on miR-361-3p expression was revealed above. Afterward, the function was taken into account. SOX4's involvements in HCC progression and the ability of miRNAs to target downstream genes have been reported previously [25, 26]. In the 3′UTR of SOX4, potential miR-361-3p binding sites were confirmed by bioinformatics tools (Figure 6(a)). The results of TCGA database revealed that SOX4 was highly expressed in HCC (Figure 6(b)). Moreover, a positive association between BAIAP2-AS1 expression and SOX4 expression was found from correlation analysis (Figure 6(c)). We also observed that both mRNA and protein levels of SOX4 expression were distinctly increased in HCC cells compared to LO2 cells (Figure 6(d)). The higher the SOX4 expression, the shorter the OS and DFS, as revealed by survival assays based on TCGA datasets (Figure 6(e)). Previously, SOX4 has been demonstrated to be an oncogene in several tumors, including HCC. Luciferase activity reporter assays were conducted for verifying whether SOX4 was a direct target of miR-361-3p. Substantial reduction in the luciferase activity was found when the cells were cotransfected with miR-361-3p mimic and the 3′UTR-SOX4-WT reporter vector. However, the cells cotransfected with the 3′UTR-SOX4-MUT reporter vector and miR-361-3p mimic in HepG2 and PLC5 cells displayed no changes in this aspect (Figure 6(f)). Also, when miR-361-3p was overexpressed, the expression of SOX4 was reduced at both protein and mRNA levels (Figure 6(g)). To study whether BAIAP2-AS1 promoted the progression of HCC via regulating miR-361-3p/SOX4 axis, we performed rescue experiments. As shown in Figure 7(a), miR-361-3p inhibitors were found to partly relieve the role of BAIAP2-AS1 knockdown in reducing SOX4's expression in HepG2 and PLC5 cells. Subsequently, we performed functional experiments, finding that the proliferative and invasive abilities of HepG2 and PLC5 which were suppressed by BAIAP2-AS1 knockdown were partly rescued by miR-361-3p downregulation (Figures 7(b)–7(e)). Moreover, we also found that SOX4 knockdown could reverse the enhanced proliferation and invasion ability of HepG2 and PLC5 cells caused by miR-361-3p inhibition (Figures 8(a)–8(c)).

## 4. Discussion

The obvious symptoms do not display in many HCC patients until an advanced stage, and an increasing occurrence of HCC was observed in Africa and China [27]. Clinical data have demonstrated that early diagnosis and individual treatments based on the advanced prediction of clinical outcomes can enhance long-term survivals of many HCC patients [28, 29]. However, sensitive biomarkers are limited. In recent years, more and more researches suggested the potential of lncRNAs to be new biomarkers for prognosis and diagnosis of HCC patients [30, 31]. Here, significantly higher-level expression of BAIAP2-AS1 was found in HCC patients, suggesting it as a potential regulator in HCC progression. However, TCGA datasets indicated that the dysregulation of BAIAP2-AS1 expression did not influence the OS and DFS of HCC patients. Thus, the results should be confirmed by larger clinical trials. Overall, our data revealed BAIAP2-AS1 to be a possible biomarker for HCC patients.

Although the abnormal expressions of many lncRNAs were observed in HCC by the use of microarray assays, the potential mechanisms remained largely unclear. Recently, just like some protein-coding genes, the transcription of lncRNAs has been reported to be modulated by transcription factors [32]. For instance, the metastasis of HCC cells was promoted by STAT3-mediated overexpression of lncRNA HOXD-AS1 [33]. Then, we searched Jaspar database and focused on E2F1 which presented a relatively higher score than other regulators. Our group also observed that E2F1 was overexpressed in HCC, which was consistent with previous studies. The role of E2F1 in regulating lncRNA transcription was also reported previously. Subsequently, we performed luciferase reporter assays and ChIP-qPCR analysis, confirming the ability of E2F1 to induce the positive transcription of BAIAP2-AS1 by binding to its promoter region. According to the combined results of the literature and this research, the abnormal activation of transcription factors may be involved in the overexpression of lncRNAs in tumor cells.

Growing studies have demonstrated that lncRNA could influence tumor cells' metastasis and proliferation. Then, we confirmed that under knockdown of BAIAP2-AS1, the proliferation and metastasis of HCC cells were suppressed. It has been reported that EMT can be an important driver of tumor growth and metastasis [34]. Then, Western blot demonstrated that knockdown of BAIAP2-AS1 can suppress the activity of EMT pathway. These findings suggested BAIAP2-AS1 played a role in promoting tumor development in HCC. To further explore the underlying molecular mechanisms, its localization in HCC cells was identified considering the dependence of lncRNA functions on its subcellular localization. Our results confirmed BAIAP2-AS1 as a cytosolic lncRNA, suggesting it can act as a microRNA sponge. The bioinformatics assays and luciferase reporter technologies suggested that BAIAP2-AS1 might bind with miR-361-3p. In many types of tumors, miR-361-3p was observed to exhibit a decreased level and act as an antioncogene, including HCC [35, 36]. In our cohort, we also observed that miR-361-3p levels were decreased in HCC, and the negative association of its levels with BAIAP2-AS1was found from the correlation analysis. Thus, we suggested BAIAP2-AS1 may exhibit its oncogenic roles via sponging miR-361-3p.

In general, as a ceRNA, the functions of lncRNAs depend on the mRNA targets of miRNAs [37]. Thus, the targeting genes of miRNAs are a crucial part of the regulatory network. Using StarBase 2.0, we found SOX4 may be a potential target of miR-361-3p. As a critical developmental transcription factor, SOX4 has been demonstrated to exhibit regulatory effects on progenitor developments, cytodifferentiation, and stemness [38, 39]. In addition, the activity of several developmental pathways is also regulated by this gene, such as TGF*β*, Wnt, and PI3K signaling [40]. In over twenty types of malignancies, SOX4 is distinctly overexpressed, and more and more functional experiments reveal SOX4 as an oncogenic gene [26]. In TCGA datasets, SOX4 was found overexpressed in HCC and a poor outcome of HCC patients was predicted. Further, luciferase reporter assays and Western blot assays identified SOX4 to be a potential target of miRNA-361-3p. To further explore whether lncRNA modulated HCC progression through the modulation of miRNA-361-3p/SOX4, we performed rescue experiments and found that when miR-361-3p decreased, the suppression of SOX4 expression mediated by the silence of BAIAP2-AS1 was reduced to a certain extent. Moreover, a series of functional experiments revealed that the suppression of HCC cell proliferation and metastasis mediated by the inhibition of BAIAP2-AS1 was relieved by miR-361-3p knockdown. In addition, SOX4 knockdown could reverse the enhanced proliferation and invasion ability of HepG2 and PLC5 cells caused by miR-361-3p inhibition. Hence, the integrated pathway of BAIAP2-AS1-miR-361-3p-SOX4 offered new insights into HCC tumorigenesis, suggesting a potential therapeutic approach via targeting the BAIAP2-AS1-miR-361-3p-SOX4 axis.

## 5. Conclusions

The results of the present research demonstrated that BAIAP2-AS1 was upregulated and activated by an E2F1 regulator in HCC cells. BAIAP2-AS1 could promote the proliferation and metastasis of HCC cells via sponging miR-361-3p and releasing SOX4. This research contributed to the development of lncRNA-directed diagnostics and therapeutics against HCC.

## Figures and Tables

**Table 1:** Primers for qPCR assays.

Names	Sequence (5′-3′)
BAIAP2-AS1: forward	GCTACCCTCGTCAGTCAAACTC
BAIAP2-AS1: reverse	GGAACGACCCACGAATCCAG
E2F1: forward	ACGCTATGAGACCTCACTGAA
E2F1: reverse	TCCTGGGTCAACCCCTCAAG
miR-361-3p: forward	GCCGCTCCCCCAGGTGTGATT
miR-361-3p: reverse	GTGCAGGGTCCGAGGT
SOX4: forward	GACCTGCTCGACCTGAACC
SOX4: reverse	CCGGGCTCGAAGTTAAAATCC
U6: forward	GCGCGTCGTGAAGCGTTC
U6: reverse	GTGCAGGGTCCGAGGT
GAPDH: forward	AGGTCGGTGTGAACGGATTTG
GAPDH: reverse	GGGGTCGTTGATGGCAACA

## Data Availability

The analyzed datasets generated during the study are available from the corresponding author on reasonable request.
